# Isolation and Characterization of the Free Phenylphosphinidene Chalcogenides C_6_H_5_P=O and C_6_H_5_P=S, the Phosphorous Analogues of Nitrosobenzene and Thionitrosobenzene

**DOI:** 10.1002/anie.202004172

**Published:** 2020-05-08

**Authors:** Artur Mardyukov, Felix Keul, Peter R. Schreiner

**Affiliations:** ^1^ Institute of Organic Chemistry Justus Liebig University Heinrich-Buff-Ring 17 35392 Giessen Germany

**Keywords:** matrix isolation, phosphinidene oxide, phosphinidene sulfide, photochemistry

## Abstract

The structures and reactivities of organic phosphinidene chalcogenides have been mainly inferred from trapping or complexation experiments. Phosphinidene chalcogenide derivatives appear to be an elusive family of molecules that have been suggested as reactive intermediates in multiple organophosphorus reactions. The quest to isolate “free” phosphinidene chalcogenides remains a challenge in the field. Here, we present the synthesis, IR, and UV/Vis spectroscopic identification of hitherto elusive phenylphosphinidene oxide and phenylphosphinidene sulfide from the corresponding phosphonic diazide precursors. We isolated these higher congeners of nitroso‐ and thionitrosobenzene in argon matrices at 10 K. The spectral assignments are supported by B3LYP/6–311++G(3df,3pd) and MP2/cc‐pVTZ computations.

## Graphical abstract

**There they are**! Phenylphosphinidene oxide and phenylphosphinidene sulphide can readily be prepared through photoinduced fragmentation of their corresponding phosphonic diazide precursors. They were identified using IR and UV/Vis spectroscopies in combination with computational methods.

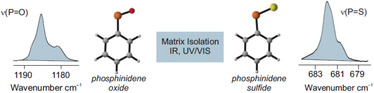

While nitrosobenzenes are common, highly reactive reagents in organic chemistry,^1^, ^2^ their heavier isosteres based on phosphorus and sulfur remain unknown. Phosphinidene chalcogenides (R−P=E; E=O, S, Se) are reactive species that are isolobal with heteroatom‐substituted singlet carbenes (Scheme 1 B),^3^, ^4^, ^5^ but they have only rarely been observed directly.^6^, ^7^, ^8^ The closely related thionitrosobenzenes are also unknown in free form and are expected to be highly unstable.^9^, ^10^, ^11^ Despite considerable efforts, phosphinidene chalcogenides are only known as transient species in solution, and their existence can be inferred from product‐analysis studies and complexation experiments.^3^, ^12^, ^13^, ^14^, ^15^, ^16^ These transient species are very reactive toward many (organic) molecules and have thus been used as in‐situ reagents for phosphorus‐ and chalcogen‐ring formation.^16^, ^17^, ^18^, ^19^ Product analyses from trapping experiments revealed that R−P=E species undergo cycloaddition reactions along with the polarized phosphorus–chalcogen double bond either showing as [4+1] carbene‐like reactivity^4^, ^20^, ^21^, ^22^, ^23^ or [4+2] olefin‐like reactivity.^15^, ^24^ Very recently, Cummins and co‐workers reported an elegant approach to generate *tert*‐butylphosphinidene sulphide (*t*BuP=S) in solution under mild conditions using anthracene (C_14_H_10_) extrusion for the release of highly reactive *t*BuP=S in situ, which undergoes a Diels–Alder reaction with dienes.^25^ In 2017, Graham et al. reported the synthesis of four‐membered phosphorus–chalcogen (RPE)_2_ heterocycles (E=S, Se);^12^ subsequent reactions with N‐heterocyclic carbenes (NHCs) resulted in base‐stabilized phosphinidene sulfides. Schmidpeter and co‐workers^26^ prepared stable monomeric phosphorous monochalcogenides without bulky or intramolecularly coordinating substituents, which are stabilized through conjugation with a triphenylphosphoniumylidyl moiety (therefore named ylidylphosphorchalcogenides) and large contributions of zwitterionic resonance structures. Product studies suggested the transient generation of phosphinidene chalcogenides (thermally or photochemically) from various precursors,^4^, ^15^, ^27^ such as phospholene, phosphirane, or phosphanorbornadiene chalcogenides,^28^, ^29^, ^30^ and starting materials containing four‐membered rings with a P_2_E_2_ core (E=S, Se).^3^, ^12^

**Scheme 1:**
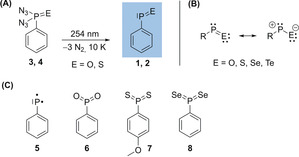
A) Photochemical generation of **1** and **2**. B) Resonance structures of the phosphinidene chalcogenides. C) Triplet phosphinidene (**5**), phenyldioxophosphorane (**6**), and previously prepared heavier congeners of **6**, namely **7** and **8**.

The direct spectroscopic observation of phosphinidene chalcogenides, R−P=E, is extremely scarce. The stabilization of R−P=E species can be achieved through coordination to metal centers^31^, ^32^ or by using bulky substituents.^33^, ^34^ However, for E=O, only a few transition‐metal complexes containing an R−P=O moiety have been isolated.^32^, ^35^ Parent H−P=O has been identified as one of the emitting species in the chemiluminescence of white phosphorus and in the oxidation of phosphine.^36^ The spectroscopy and structure of H−P=O has been intensively studied.^37^, ^38^ The heavier congener H−P=S has been detected with neutralization–reionization mass spectrometry^39^ and rotational spectroscopy.^40^ Only in 2019, CH_3_−P=O was generated and efficiently trapped in argon matrices through photolysis or flash vacuum pyrolysis of methylphosphoryl diazide CH_3_P(O)(N_3_)_2_.^41^

As found for phosphinidene oxides and sulfides, dioxophosphoranes (phosphinidene dioxides, R−PO_2_) and thiooxophosphoranes (R−PS_2_) are unstable molecules thought to be generated in the thermolysis of suitable organophosphorus precursors.^42^, ^43^, ^44^ These transient species are highly electrophilic at the phosphorus atom and have thus found use as efficient phosphorylating agents. Recently, we reported the synthesis of previously elusive phenyldioxophosphorane (**6**, PhPO_2_), the phosphorus analogue of nitrobenzene, under matrix‐isolation conditions through the reaction of triplet phenylphosphinidene (**5**) with triplet molecular oxygen (^3^P‐O_2_).^45^ The heavier congeners of **7**, namely (4‐methoxy)phenyl phosphine disulphide (**6**)^46^ and phenyl phosphine diselenide (**8**)^47^ (the monomeric forms of Lawesson's and Woollins’ reagents, respectively), have been isolated and characterized spectroscopically as well (Scheme 1 C). Following our studies on the synthesis and reactivity of transient organophosphorus species including PhP,^45^ PhPO_2_,^45^ PhPCO,^48^ PhPS_2_,^46^ and PhPSe_2_,^47^ we report herein the first spectroscopic evidence of hitherto unknown “free” (that is, uncomplexed) phenylphosphinidene oxide Ph−P=O (**1**) and phenylphosphinidene sulfide Ph−P=S (**2**) by means of IR and UV/Vis spectroscopy (Scheme 1 A).

Phosphonic diazides represent potentially useful precursors for the generation of free phosphinidene chalcogenides that can be activated either thermally or photochemically, and we have chosen the UV photolysis of phenylphosphoryl diazide (**3**) in an argon matrix at 10 K with N_2_ as the only IR‐invisible byproduct. The IR spectrum of **3** is characterized by an intense group of signals centered at 2153 cm^−1^ (Figure 1). Irradiation (*λ*=254 nm) of matrices containing **3** results in very rapid and complete disappearance of its IR signals. From a number of experiments, we identify a set of IR bands (Figure 1 and Table S1, Supporting Information), the most prominent at 1185, 1098, 741, 689, and 462 cm^−1^, that show identical growth behavior upon photolysis. These signals are assigned to **1** based on comparison with B3LYP/6–311++G(3df,3pd) computations. The strong IR bands at 1185 and 1098 cm^−1^ are attributed to the P=O stretching and C−H deformation modes. The observed splitting of the vibrational bands is due to different trapping sites in the argon matrix. The measured P=O stretching frequency agrees well with a previously observed IR band at 1203 cm^−1^ for the stabilized neutral 2,4,6‐tri‐*tert*‐butylphenylphosphinidine oxide.^33^, ^35^ The bands at 741, 706, and 689 cm^−1^ are thus assigned to the C−H out‐of‐plane vibrational modes of the phenyl ring. Overall, the observed IR vibrational bands match the computed fundamentals of **1** (Table S1) very well.

**Figure 1:**
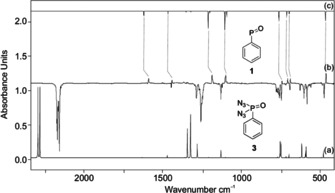
a) IR spectrum of **3** computed at B3LYP/6–311++G(3df,3pd) (unscaled). b) IR difference spectra showing the photochemistry of **3** after irradiation at *λ*=254 nm in argon at 10 K. Downward bands assigned to **3** disappear while upward bands assigned to **1** appear after 15 min irradiation time. c) IR spectrum of **1** computed at B3LYP/6–311++G(3df,3pd) (unscaled).

Photochemical decomposition of **3** with 254 nm irradiation resulted in a slight yellow coloration of the matrix, which was colorless initially. The UV/Vis spectral analysis of the 254 nm photolysis of **3** shows that its strong absorptions at 192 and 217 nm gradually decrease while new absorptions grow. The spectrum obtained after complete decomposition of azide precursor **3** reveals strong absorptions at 192, 219, and 277 nm, a weak absorption at 337 nm, as well as several transitions with pronounced vibrational progressions extending from 430 nm up to 490 nm (Figure 2). Computational analysis using time‐dependent density functional theory (TD‐B3LYP/6–311++G(3df,3pd)) of the excitations of **1** exhibit a strong transition at 284 nm (*f*=0.222) and two weak transitions at 314 nm (*f*=0.011) and 474 nm (*f*=0.004) in good agreement with the experimentally observed UV/Vis spectrum (Figure 2). Likewise, in our earlier studies on reactive organophosphorus species,^45^, ^46^, ^47^, ^48^ the inspection of the molecular orbitals of **1** reveals that the weak absorption band in the visible region at 460 nm corresponds an n→π* transition, while the strong band at 277 nm is a π→π* transition.

**Figure 2:**
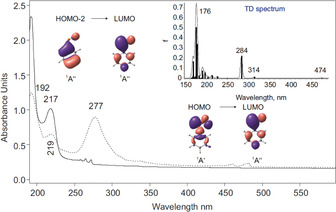
Solid: UV/Vis spectrum of **3** isolated at 10 K in Ar. Dashed: UV/Vis spectrum of **1** at 10 K; the photochemistry of **3** after irradiation at *λ*=254 nm in Ar at 10 K. Inset: Computed TD‐B3LYP/6–311++G(3df,3pd) spectrum of **1**.

Following the route for the synthesis of **1**, we prepared **2** from the azide **4** by UV irradiation. Photolysis (*λ*=254 nm) of matrix‐isolated **4** in solid argon for 30 min at 10 K resulted in the complete disappearance of its IR bands and the appearance of a new set of IR bands. New strong bands appeared at 1185, 1084, 743, 706, 682, and 416 cm^−1^ upon UV irradiation (Figure 3 and Table S2), in good agreement with the computed values. For example, the band at 1185 and 1084 are assigned to the C−H deformation modes in **2**. The bands at 743, 706, and 685 cm^−1^ are assigned to the C−H out‐of‐plane vibrational modes of the phenyl ring. The strong band at 682 cm^−1^ is assigned to the P=S stretching mode of **2**; as expected, it absorbs at a lower frequency compared to **1** due to the considerably longer P=S bond (see below). The nature of the P=S bond in **1** can also be judged by comparison with the P=S stretching modes in related compounds. Several unstable thiophosphines (X−P=S; X=Br, F) have been characterized spectroscopically either in the gas phase or have been isolated in argon matrices.^49^ F−P=S shows an intense P=S stretching frequency at 720 cm^−1^,^7^ whereas Br−P=S absorbs at 712 cm^−1^.^8^ With the help of the computations, the other IR bands of medium intensity can also be attributed to **2** (Table S2).

**Figure 3:**
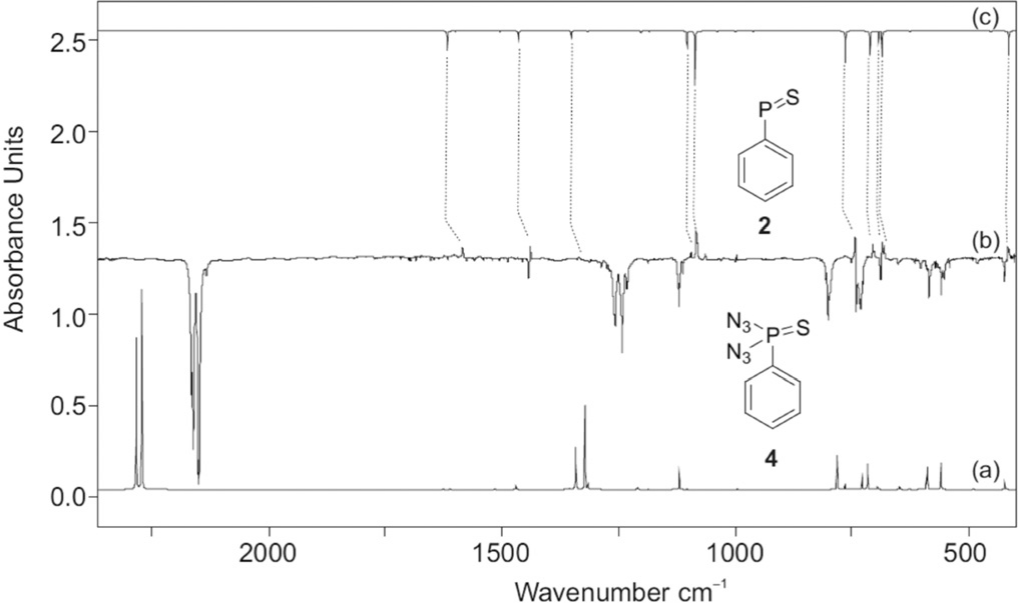
a) IR spectrum of **4** computed at B3LYP/6–311++G(3df,3pd) (unscaled). b) IR difference spectra showing the photochemistry of **4** after irradiation at *λ*=254 nm in argon at 10 K. Downward bands assigned to **4** disappear while upward bands assigned to **2** appear after 15 min irradiation time. c) IR spectrum of **2** computed at B3LYP/6–311++G(3df,3pd) (unscaled).

The photochemistry of **4** was also investigated in the UV/Vis spectral region. Under reaction conditions similar to the IR experiments described above, irradiation of an argon matrix containing **4** results in a decrease in intensity of the bands at *λ*=194 and 220 nm, assigned to **4** and formation of new strong bands at *λ*_max_=192 and *λ*_max_=337 nm assigned to **2** (Figure 4 and Figure S2). The measured UV/Vis spectrum of **2** also displays a weak absorption in the 600–730 nm range with a pronounced vibrational fine structure. Similar to **1**, all bands of **2** correlate well with the values of the electronic excitations at 191 and 218 nm (*f*=0.201 and 0.074), 331 nm (*f*=0.227), and 692 nm (*f*=0.0008) computed at TD‐B3LYP/6–311++G(3df,3pd).

**Figure 4:**
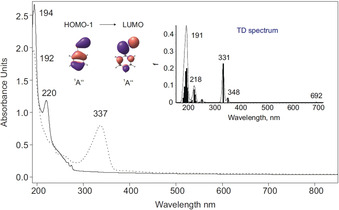
Solid: UV/Vis spectrum of **4** isolated at 10 K in Ar. Dashed: UV/Vis spectrum of **2** at 10 K; the photochemistry of **4** after irradiation at *λ*=254 nm in Ar at 10 K. Inset: Computed TD‐B3LYP/6–311++G(3df,3pd) spectrum of **2**.

Next, we compared the key geometrical and electronic parameters of the optimized structure of **1** with **2** (Figure 5 A). The P=O and P=S bond distances in **1** and **2** are 1.485 and 1.937 Å, respectively, at B3LYP/6–311++G(3df,3dp) (1.504 and 1.942 Å at MP2/cc‐pVTZ), thus showing double‐bond character. Wiberg bond indices of 1.74 and 1.54 were computed for the P=O and P=S bonds in **1** and **2**, respectively, indicating significant double‐bond character. The P=O length in **1** is comparable to the corresponding values in previously reported compounds with R−P=O ligands as well as with an anionic molybdenum‐bound phosphinidene oxide complex with a P=O bond of 1.514 Å.^32^ The P=S bond distance in **2** is only slightly shorter than the P=S bond distance of 2.028 Å in the NHC adduct of aryl phosphine sulphide.^12^ The computed C−P bond distances in **1** and **2** are 1.819 and 1.820 Å, respectively, at B3LYP/6–311++G(3df,3dp) (1.824 and 1.822 Å at MP2/cc‐pVTZ) with bond‐dissociation energies (BDE) of 73.4 and 68.5 kcal mol^−1^ (including zero‐point vibrational‐energy corrections, ZPVEs). No significant geometric differences were observed in the phenyl rings. This indicates there is negligible electronic delocalization of the P=E moiety with the phenyl ring (see below). According to our computations, **1** and **2** are planar *C*_s_‐symmetric structures with a ^1^A′ electronic ground state.

**Figure 5:**
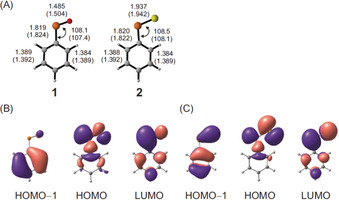
A) Selected bond lengths [Å] and angles [°] of **1** and **2** at B3LYP/6–311++G(3df,3pd). The values in parentheses were computed at MP2/cc‐pVTZ; B), C) Molecular orbitals of **1** (B) and **2** (C) at B3LYP/6–311++G(3df,3pd).

The NPA atomic charges of **1** and **2** are +0.33 and +0.07 *e* at phosphorus, while the oxygen and sulfur atoms have considerable negative charges of −0.34 and −0.33 *e*, respectively. Sulfur forms weaker and longer bonds than oxygen, owing to the larger sulfur atom with larger and more diffuse orbitals, resulting in poorer orbital overlap with the phosphorus orbitals as compared to the oxygen atom. Indeed, the HOMOs (highest occupied molecular orbitals) in **1** and **2** are in‐plane orbitals combining the σ(P−C) orbital, the P lone pair, and an in‐plane p‐orbital of the chalcogen. Carbene‐like features are evident from the finding that the HOMO entails the P lone pair and the LUMO the empty p_*z*_ orbital on phosphorus; the isolobal analogy to a singlet carbene holds.^4^ The HOMO−1 of **1** is mainly localized over the phenyl ring, while in **2**, the HOMO−1 displays pronounced π‐bonding between the phosphorus and sulfur atoms (Figure 5 B,C). When sulfur is present instead of oxygen, there is a higher destabilization of the HOMO energy, leading to a smaller HOMO–LUMO gap; this indicates that **2** is more basic than **1**. Thus, the estimated Δ*E*=*E*_HOMO_−*E*_LUMO_ value for **2** (0.10 eV) is smaller than that of **1** (0.14 eV). The resulting smaller HOMO–LUMO gap correlates well with the UV/Vis absorption maxima of **2**, which is red‐shifted for **2** (*λ*_max_≈660 nm) compared to **1** (*λ*_max_≈460 nm).

In summary, we present the generation and isolation of phenylphosphinide oxide and phenylphosphinidene sulfide using a combination of photolysis, matrix‐isolation IR, and UV/Vis spectroscopic methods as well as quantum‐chemical computations. The bis‐azide precursors are easy to prepare and readily undergo thermal or photochemical decomposition to give **1** and **2**. The facile generation of **1** and **2** opens the door for further experimental studies on their (photo)reactivity.

## Conflict of interest

The authors declare no conflict of interest.

## Supporting information

As a service to our authors and readers, this journal provides supporting information supplied by the authors. Such materials are peer reviewed and may be re‐organized for online delivery, but are not copy‐edited or typeset. Technical support issues arising from supporting information (other than missing files) should be addressed to the authors.

Supplementary
