# Supplementary material for: Isolation and Characterization of the Free Phenylphosphinidene Chalcogenides C6H5P=O and C6H5P=S, the Phosphorous Analogues of Nitrosobenzene and Thionitrosobenzene
Source: Angew Chem Int Ed Engl. 2020 May 8;59(30):12445–9. doi: 10.1002/anie.202004172 (PMC7384188; doi:10.1002/anie.202004172)
Supplement: Supplementary file 1 — Supplementary [file ANIE-59-12445-s001.pdf]

Angewandte Chemie
Eine Zeitschrift der Gesellschaft Deutscher Chemiker

Supporting Information

Isolation and Characterization of the Free Phenylphosphinidene Chalcogenides $C_6H_5P{=}O$ and $C_6H_5P{=}S$, the Phosphorous Analogues of Nitrosobenzene and Thionitrosobenzene

Artur Mardyukov, Felix Keul, and Peter R. Schreiner**

anie_202004172_sm_miscellaneous_information.pdf

Table of Contents

Table of Contents 2
Experimental Procedures 2
Computations. 2
Figure S1 4
Figure S2. 5
Table S1. 6
Table S2. 7
References 7
Geometric Structures and Electronic energies 8

Experimental Procedures

Matrix Apparatus Design. For the matrix isolation studies, we used an APD Cryogenics HC-2 cryostat with a closed-cycle refrigerator system, equipped with an inner CsI window for IR measurements. Spectra were recorded with a Bruker IFS 55 FT-IR spectrometer with a spectral range of 4500−400 cm^{-1} and a resolution of 0.7 cm^{-1} and UV/Vis spectra were recorded with a JASCO V-670 spectrophotometer equipped with an inner sapphire window. A high-pressure mercury lamp (HBO 200, Osram) with a monochromator (Bausch & Lomb) was used for irradiation.

For the combination of high-vacuum flash pyrolysis with matrix isolation, we employed a small, home-built, water-cooled oven, which was directly connected to the vacuum shroud of the cryostat. The pyrolysis zone consisted of an empty quartz tube with an inner diameter of 8 mm, which was resistively heated over a length of 50 mm by a coaxial wire. The temperature was monitored with a NiCr−Ni thermocouple. Compounds **3** and **4** (Sigma-Aldrich) were evaporated (10 °C) from a storage bulb into the quartz pyrolysis tube. At a distance of approximately 50 mm, all pyrolysis products were co-condensed with a large excess of argon (typically 60−120 mbar from a 2000 mL storage bulb) on the surface of the matrix window at 10 K (20 K). Several experiments with pyrolysis temperatures ranging from 200 to 700 °C were performed in order to determine the optimal pyrolysis conditions.

Computations. All geometries were optimized and characterized as minima or transition structures by means of analytical harmonic vibrational frequency computations at the B3LYP/6-311++G(3df,3pd) level of theory.[1-3] All computations were performed with the Gaussian16 program.[4]

Synthesis of phenyl phosphonic diazide

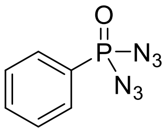

To a solution of 95 µL (0.672 mmol, 1.00 equiv.) phenyl phosphonic dichloride in 1 mL dry THF 535 µL (4.03 mmol, 6.00 equiv.) TMS-N_3 was added under inert conditions. The solution was refluxed. After 24 h the temperature was decreased to 40 °C and the solvents were evaporated at 0.2 mbar. A colorless liquid was obtained.
^{1}H-NMR (400 MHz, MeCN-d_3): δ/ppm = 7.93 – 7.83 (m, 2H); 7.79 – 7.71 (m, 1H); 7.66 – 7.58 (m, 2H). **^{13}C-NMR (101 MHz, MeCN-d_3):** δ/ppm = 135.5 (d, 1C); 132.0 (d, 2C); 130.3 (d, 2C); 127.9 (d, 1C). **^{31}P-NMR (400 MHz, MeCN-d_3):** δ/ppm = 18.44.

Synthesis of phenyl thiophosphonic diazide

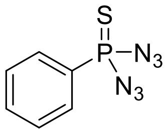

To a solution of 101 µL (0.672 mmol, 1.00 equiv.) phenyl phosphonic dichloride in 1 mL dry THF 1.07 mL (8.06 mmol, 12.0 equiv.) TMS-N_3 was added under inert conditions. The solution was refluxed for 48 h. The temperature was decreased to 40 °C and the solvents were evaporated at 0.2 mbar. A yellowish liquid was obtained.
^{1}H-NMR (400 MHz, MeCN-d_3): δ/ppm = 8.00 – 7.88 (m, 2H); 7.73 – 7.65 (m, 1H); 7.62 – 7.53 (m, 2H). **^{13}C-NMR (101 MHz, MeCN-d_3):** □/ppm = 134.9 (d, 1C); 131.7 (d, 1C); 131.1 (d, 2C); 129.9 (d, 2C). **^{31}P-NMR (400 MHz, MeCN-d_3):** δ/ppm = 73.70.

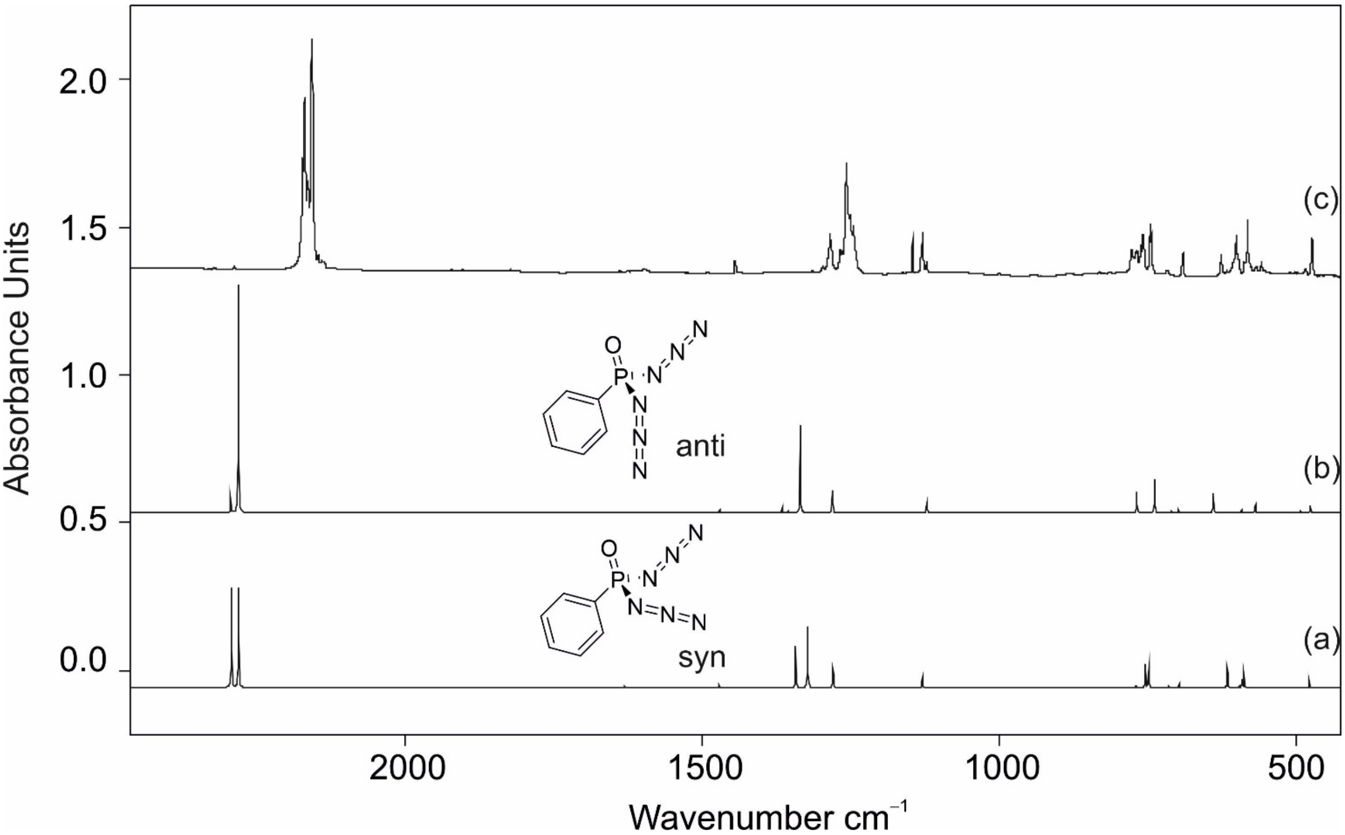

Figure S1. (a) IR spectrum of syn-**3** computed at B3LYP/6-311++G(3df,3pd) (unscaled). (b) IR spectrum of anti-**3** computed at B3LYP/6-311++G(3df,3pd) (unscaled). (c) IR spectrum of matrix-isolated **3** in argon at 10 K.

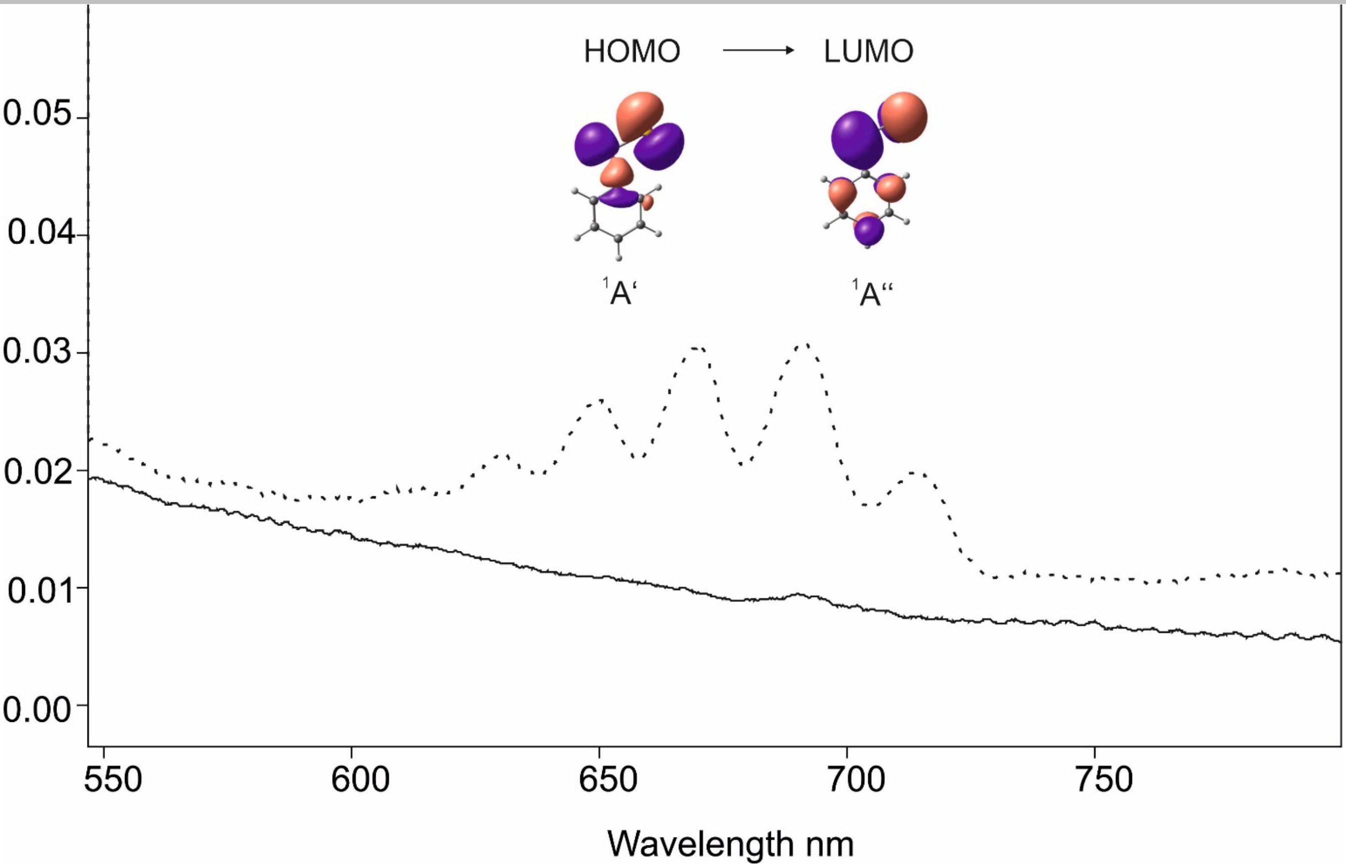

Figure S2. Solid: UV/Vis spectrum of **4** isolated at 10 K in Ar. Dashed: UV/Vis spectrum of **2** at 10 K; the photochemistry of **4** after irradiation at λ = 254 nm in Ar at 10 K.

Table S1. Experimental (Ar matrix, 10 K) and computed IR frequencies of **1**, band origins in cm^{-1}, computed intensities (km mol^{-1}) in parentheses.

Mode	Computed[a]	Ar, 10 K[b]	Symm	Assignment
28	1619 (35.6)	1587 (m)	A’	C=C str.
27	1602 (0.2)	-	A’	C=C str.
26	1505 (1.4)	-	A’	CH def.
25	1465 (17.4)	1439 (m)	A’	CH def.
24	1351 (6.0)	1345(w)	A’	CH def. / C=C str.
23	1320 (4.6)	1311 (w)	A’	ring distortion
22	1210 (107.9)	1185 (s)	A’	P=O str.
21	1199 (8.0)	1181 (m)	A’	CH def.
20	1185 (0.7)	-	A’	CH def.
19	1105 (107.3)	1098 (s)	A’	CH def.
18	1093 (36.6)	1091 (w)	A’	ring distortion
17	1038 (0.6)	-	A’	ring distortion
16	1024 (0.0)	-	A’’	CH o.o.p. def.
15	1010 (0.0)	-	A’’	CH o.o.p. def.
14	995 (0.7)	-	A’	ring breathing
13	963 (1.4)	-	A’’	CH o.o.p. def.
12	876 (0.0)	-	A’’	CH o.o.p. def.
11	762 (52.4)	741(s)	A’’	CH o.o.p. def.
10	711 (25.2)	706 (s)	A’	ring distortion
9	696 (23.1)	689 (s)	A’’	ring breathing
8	627 (0.1)	-	A’	ring distortion
7	466 (74.5)	462 (s)	A’	CPO def.
6	445 (1.8)	-	A’’	ring breathing
5	405 (0.1)	-	A’’	ring distortion

[a]M06-2x/6-311++G(3df,3pd), harmonic approximation, unscaled frequencies, intensities (in parentheses) in km mol^{-1}. [b] Experiment: argon matrix, 10 K.; approximate relative intensities (w: weak, m: medium, s: strong).

Table S2. Experimental (Ar matrix, 10 K) and computed IR frequencies of **3**, band origins in cm^{-1}, computed intensities (km mol^{-1}) in parentheses.

Mode	Computed[a]	Ar, 10 K[b]	Symm	Assignment
28	1618 (28.9)	1585 (m)	A’	C=C str.
27	1600 (0.5)	-	A’	C=C str.
26	1504 (0.4)	-	A’	CH def.
25	1465 (17.3)	1439 (m)	A’	CH def.
24	1352 (8.3)	1334(w)	A’	CH def. / C=C str.
23	1317 (2.3)	-	A’	ring distortion
22	1203 (4.7)	1185 (s)	A’	CH def.
21	1185 (0.4)	1181 (m)	A’	CH def.
20	1104 (30.5)	1095 (w)	A’	CH def.
19	1087 (84.8)	1084 (s)	A’	CH def.
18	1040 (1.2)	1091 (w)	A’	ring distortion
17	1022 (0.0)	-	A’’	CH o.o.p. def.
16	1011 (0.0)	-	A’’	CH o.o.p. def.
15	1000 (1.8)	-	A’	ring distortion
14	963 (1.0)	-	A’’	CH o.o.p. def.
13	873 (0.0)	-	A’’	CH o.o.p. def.
12	765 (49.6)	743 (s)	A’’	CH o.o.p. def.
11	712 (44.5)	706(s)	A’	ring breathing
10	694 (22.0)	685 (s)	A’	CH o.o.p. def.
9	686 (38.8)	682 (s)	A’’	P=S str.
8	627 (1.5)	-	A’	ring distortion
7	454 (1.9)	-	A’’	ring breathing
6	415 (38.1)	416 (s)	A’	CPS def.
5	406 (0.0)	-	A’’	ring breathing

[a] B3LYP/6-311++G(3df,3pd), harmonic approximation, unscaled frequencies, intensities (in parentheses) in km mol^{-1}. [b] Experiment: argon matrix, 10 K.; approximate relative intensities (w: weak, m: moderate, s: strong).

References

(1) Becke, A. D. Density-functional exchange-energy approximation with correct asymptotic behavior. Phys. Rev. A: Gen. Phys. 1988, 38, 3098-3100.

(2) Lee, C.; Yang, W.; Parr, R. G. Development of the Colle-Salvetti correlation-energy formula into a functional of the electron density. Phys. Rev. B: Condens. Matter 1988, 37, 785-789.

(3) Miehlich, B.; Savin, A.; Stoll, H.; Preuss, H. Results obtained with the correlation energy density functionals of Becke and Lee, Yang and Parr. Chem. Phys. Lett. 1989, 157, 200-206.

(4) Frisch, M. J.; Trucks, G. W.; Schlegel, H. B.; Scuseria, G. E.; Robb, M. A.; Cheeseman, J. R.; Scalmani, G.; Barone, V.; Petersson, G. A.; Nakatsuji, H.; Li, X.; Caricato, M.; Marenich, A.; Bloino, J.; Janesko, B. G.; Gomperts, R.; Mennucci, B.; Hratchian, H. P.; Ortiz, J. V.; Izmaylov, A. F.; Sonnenberg, J. L.; Williams-Young, D.; Ding, F.; Lipparini, F.; Egidi, F.; Goings, J.; Peng, B.; Petrone, A.; Henderson, T.; Ranasinghe, D.; Zakrzewski, V. G.; Gao, J.; Rega, N.; Zheng, G.; Liang, W.; Hada, M.; Ehara, M.; Toyota, K.; Fukuda, R.; Hasegawa, J.; Ishida, M.; Nakajima, T.; Honda, Y.; Kitao, O.; Nakai, H.; Vreven, T.; Throssell, K.; Montgomery, J., J. A.; Peralta, J. E.; Ogliaro, F.; Bearpark, M.; Heyd, J. J.; Brothers, E.; Kudin, K. N.; Staroverov, V. N.; Keith, T.; Kobayashi, R.; Normand, J.; Raghavachari, K.; Rendell, A.; Burant, J. C.; Iyengar, S. S.; Tomasi, J.; Cossi, M.; Millam, J. M.; Klene, M.; Adamo, C.; Cammi, R.; Ochterski, J. W.; Martin, R. L.; Morokuma, K.; Farkas, O.; Foresman, J. B.; Fox, D. J. Gaussian, Inc., Wallingford CT, 2016.

(5) M. Jesberger, T. P. Davis and L. Barner, Synthesis, 2003, 1929-1958.

(6) Legnani, L. Toma, P. Caramella, M. A. Chiacchio, S. Giofre, I. Delso, T. Tejero and P. Merino, J. Org. Chem., 2016, 81, 7733-7740.

SUPPORTING INFORMATION

Geometric Structures and Electronic energies

1: (C_s point group)

0 1
6 1.552991000 -2.050977000 0.000000000
6 0.160904000 -2.150205000 0.000000000
6 -0.613428000 -1.002132000 0.000000000
6 0.000000000 0.257624000 0.000000000
6 1.397236000 0.344941000 0.000000000
6 2.173938000 -0.806606000 0.000000000
1 2.154262000 -2.950558000 0.000000000
1 -0.309868000 -3.124112000 0.000000000
1 -1.694112000 -1.057437000 0.000000000
1 1.873726000 1.318950000 0.000000000
1 3.253184000 -0.737681000 0.000000000
15 -0.944936000 1.812982000 0.000000000
8 -2.391625000 1.475030000 0.000000000
E[B3LYP] = -648.303217
ZPVE[B3LYP] = 0.0941102

3: (C_1 point group)
0 1

6 -3.807416000 -0.283291000 -0.272348000
6 -3.300497000 0.206070000 0.925085000
6 -1.926511000 0.271634000 1.123452000
6 -1.059193000 -0.156597000 0.117316000
6 -1.571096000 -0.646411000 -1.087106000
6 -2.943320000 -0.709822000 -1.277482000
1 -4.877062000 -0.334615000 -0.424815000
1 -3.971942000 0.534779000 1.706216000
1 -1.518627000 0.644678000 2.052270000
1 -0.901176000 -0.979618000 -1.867422000
1 -3.339826000 -1.091935000 -2.208060000
15 0.699585000 -0.018691000 0.420374000
7 1.252382000 -1.545318000 -0.065396000
7 1.357240000 0.924659000 -0.851989000
7 2.459301000 -1.791370000 -0.078889000
7 3.531876000 -2.116963000 -0.123286000
7 1.537699000 2.129848000 -0.674084000
7 1.742551000 3.231563000 -0.608370000
8 1.074464000 0.448830000 1.759598000
E[B3LYP] = -976.851743
ZPVE[B3LYP] = 0.1234277

3a: (C_1 point group)
0 1

6 -3.583087000 -0.000018000 -0.120786000
6 -2.976612000 -0.000014000 1.129387000
6 -1.590444000 -0.000007000 1.230591000

6	-0.809008000	-0.000004000	0.073432000
6	-1.422711000	-0.000008000	-1.183053000
6	-2.806521000	-0.000015000	-1.276598000
1	-4.662024000	-0.000024000	-0.197418000
1	-3.580297000	-0.000016000	2.026473000
1	-1.105322000	-0.000003000	2.196319000
1	-0.820496000	-0.000006000	-2.081446000
1	-3.280030000	-0.000018000	-2.248732000
15	0.980915000	0.000004000	0.253608000
7	1.571635000	-1.242822000	-0.744142000
7	1.571624000	1.242838000	-0.744139000
7	1.564666000	-2.401769000	-0.331819000
7	1.608246000	-3.488812000	-0.053832000
7	1.564632000	2.401786000	-0.331819000
7	1.608192000	3.488830000	-0.053833000
8	1.430222000	0.000005000	1.649991000

E[B3LYP] = -976.851045
ZPVE[B3LYP] = 0.1234805

2: (C_s point group)

0 1

6	2.587389000	1.387223000	0.000000000
6	2.391003000	0.005549000	0.000000000
6	1.107628000	-0.512863000	0.000000000
6	0.000000000	0.344258000	0.000000000
6	0.210734000	1.730742000	0.000000000
6	1.498514000	2.250906000	0.000000000
1	3.592593000	1.787377000	0.000000000
1	3.243616000	-0.660203000	0.000000000
1	0.942798000	-1.582262000	0.000000000
1	-0.641878000	2.399491000	0.000000000
1	1.652296000	3.321345000	0.000000000
15	-1.743552000	-0.178833000	0.000000000
16	-1.837984000	-2.113634000	0.000000000

E[B3LYP] = -971.276496
ZPVE[B3LYP] = 0.0926588

4: (C_1 point group)
0 1

6	-3.970784000	-0.155547000	-0.151490000
6	-3.367378000	0.377273000	0.980422000
6	-1.981556000	0.405103000	1.083464000
6	-1.198697000	-0.103780000	0.048925000
6	-1.806561000	-0.637838000	-1.091275000
6	-3.189774000	-0.662878000	-1.186390000
1	-5.049357000	-0.177214000	-0.229758000
1	-3.972176000	0.770279000	1.785917000
1	-1.503242000	0.814107000	1.962135000
1	-1.203363000	-1.033089000	-1.896117000
1	-3.659035000	-1.078433000	-2.067533000

15	0.596809000	-0.035986000	0.183099000
7	0.979539000	-1.637768000	-0.278617000
7	1.084934000	0.783558000	-1.256466000
7	2.137882000	-2.040808000	-0.175549000
7	3.159401000	-2.500641000	-0.105763000
7	1.613276000	1.890606000	-1.180576000
7	2.106720000	2.899154000	-1.201956000
16	1.371954000	0.634464000	1.811838000

E[B3LYP] = -1299.811831
ZPVE[B3LYP] = 0.121076
